# Metabolism in Typhoid

**Published:** 1915-12

**Authors:** 


**Metabolism in Typhoid.** Coleman and Dubois, Arch of Int. Med., May 1915. Direct and indirect calorimetry in 61 cases, agreed within 2.2%. The same degree of oxidation and the same end-products occur as in health. It is stated that the rectal temperature does not give as good an idea of the average temperature of the body as surface thermometers, well covered. At the height of the fever, the heat production is 40-50% above the normal. Heat elimination is also increased. Fat may be stored while the patient is losing weight through the toxic destruction of body protein.

